# Warts and the X-Rays

**Published:** 1908-08-29

**Authors:** 


### NOTES ON TREATMENT.

### WARTS AND THE X-RAYS.

There are a great many warts, especially in children, that require no treatment at all, for they cause little or no discomfort, and by the age at which personal appearance becomes a prime consideration they very often disappear spontaneously. Nevertheless, there are patients who suffer from warts to such a degree that the doctor is asked to remove them, and it is worth while to know that the very simplest way of getting rid of a wart is by a single application of the *x*-rays. The mode of application in the case of warts is very similar to that employed in ringworm. The wart does not fall off during the actual application, but within a week or ten days afterwards it simply drops off, leaving smooth and healthy skin behind it. The time occupied by each sitting is something between fifteen and thirty minutes, and no dressings or other application are required. The procedure gives a minimum amount of trouble to the patient, a maximum of certainty of immediate cure, and no scarring.

One kind of wart which is particularly annoying to its possessor is that which grows upon the scalp amongst the hair. Every time the head is combed the teeth of the comb may catch the wart, and give rise not only to acute pain, but also not infrequently to bleeding. These warts may occur in people who are quite grown up or even past middle life. It is as easily cured by an application of the *x*-rays as are the warts upon juvenile hands.

